# Supplementary figures and images for: The effect of a telephone-based intervention on physical activity after stroke
Source: PLoS One. 2022 Oct 20;17(10):e0276316. doi: 10.1371/journal.pone.0276316 (PMC9584526; doi:10.1371/journal.pone.0276316)

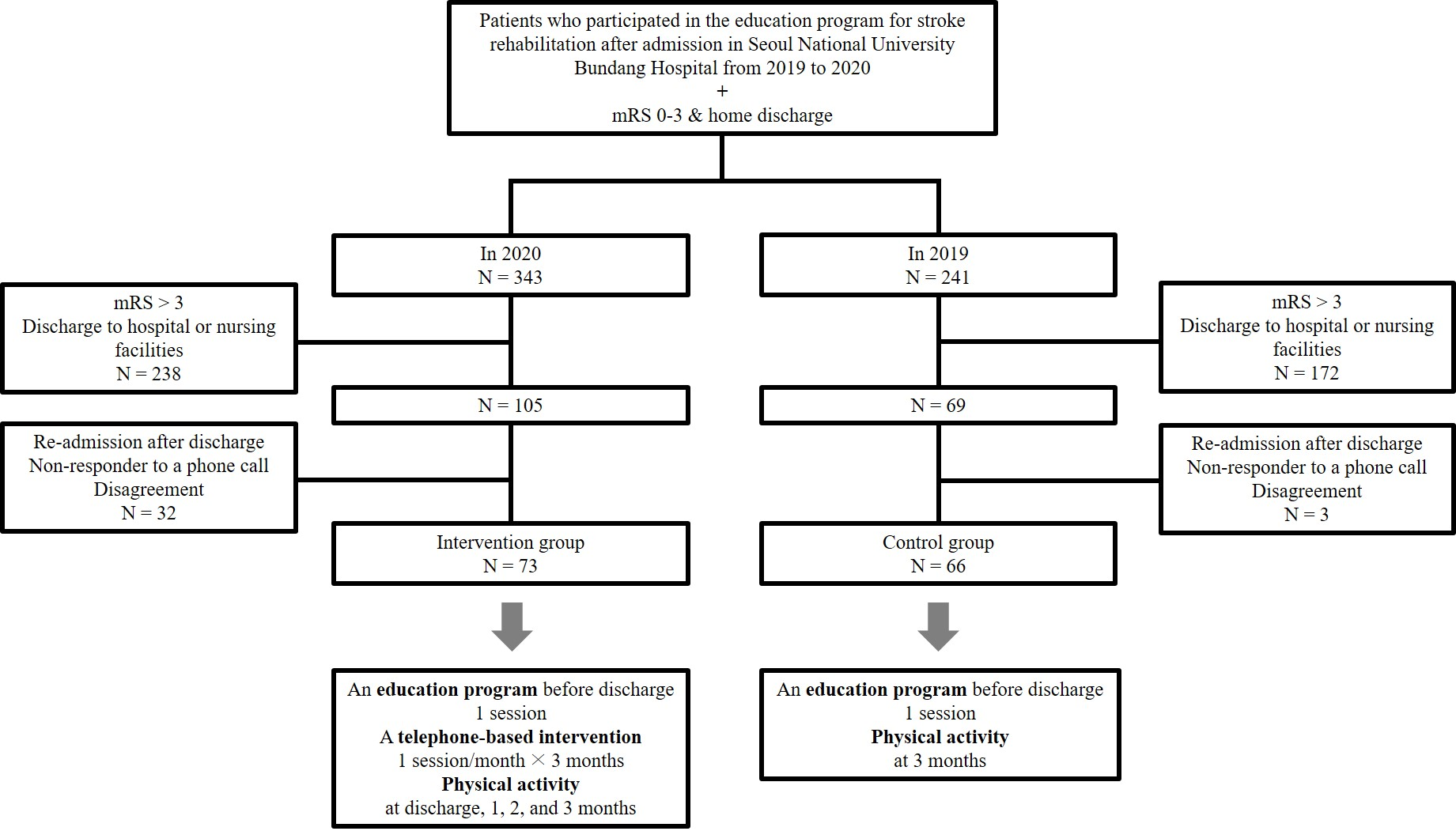

Supplement: S1 Fig — (TIF) [file pone.0276316.s001.tif]
