# Supplementary material for: The effect of a telephone-based intervention on physical activity after stroke
Source: PLoS One. 2022 Oct 20;17(10):e0276316. doi: 10.1371/journal.pone.0276316 (PMC9584526; doi:10.1371/journal.pone.0276316)
Supplement: S1 Table — (DOCX) [file pone.0276316.s002.docx]

**S1 Table. Questions frequently used in the intervention.**

| **Class** | **Questions** | **Possible options** |
| --- | --- | --- |
| Awareness | What do you think about your current level of physical activity | Provide information about whether the current level of physical activity is sufficient or not  Educate about the need to increase the current level of physical activity |
|  | How important do you think physical activity is for prognosis | Explain the importance and reason for increasing physical activity after stroke (risk factor management, functional outcomes, quality of life, etc.) |
| Physical activity  preference | What kind of exercises did you usually do before stroke | Encourage to continue those exercises if possible |
|  | Which do you like better walking, running, or ball games such as badminton, tennis and table tennis | Help to find favourable types of physical activities and encourage to continue those activities |
| Facilitators | Who can help you when going outdoor | Provide information about stroke survivors’ meeting and communities if needed |
|  | Are there any sports facilities nearby | Introduce rehabilitation facilities or sports center |
| Barriers | Are there any difficulties while you are engaged in physical activities | Provide counselling tailored to each difficulty (Adjust the intensity of physical activity to a light to moderate degree of shortness, visit the clinic if symptoms such as dizziness, pain, or severe fatigue are present, etc.) |
|  | Do you have troubles regarding fatigue, sleep, or pain | Recommend visiting the clinic if activities of daily living are impaired |
|  | Do you feel depressed or less motivated? | Recommend consulting the psychiatry department if symptoms are prominent |
| Red flag signs | Do you have any weakness, numbness, dizziness, or difficulty in speaking or understanding? | Offer an immediate neurological consultation |
