# Supplementary material for: The effect of a telephone-based intervention on physical activity after stroke
Source: PLoS One. 2022 Oct 20;17(10):e0276316. doi: 10.1371/journal.pone.0276316 (PMC9584526; doi:10.1371/journal.pone.0276316)
Supplement: S2 Table — (DOCX) [file pone.0276316.s003.docx]

**S2 Table. Physical activity categorization.**

| Health enhancing  physical activity | 1) Vigorous-intensity activity on at least 3 days achieving a minimum of at least 1500 MET-min/week  **OR**  2) 7 or more days of any combination of walking, moderate-intensity or vigorous-intensity activities achieving a minimum of at least 3000 MET-min/week |
| --- | --- |
| Minimally active | 1) 3 or more days of vigorous activity of at least 20 minutes per day  **OR**  2) 5 or more days of moderate-intensity activity or walking of at least 30 minutes per day  **OR**  3) 5 or more days of any combination of walking, moderate-intensity or vigorous-intensity activities achieving a minimum of at least 600 MET-min/week |
| Inactive | Others |
