# Supplementary material for: The effect of a telephone-based intervention on physical activity after stroke
Source: PLoS One. 2022 Oct 20;17(10):e0276316. doi: 10.1371/journal.pone.0276316 (PMC9584526; doi:10.1371/journal.pone.0276316)
Supplement: S3 Table — (DOCX) [file pone.0276316.s004.docx]

**S3 Table. Comparison of baseline characteristics between active and inactive group three months after discharge.**

|  | All (n = 139) | | | Intervention group (n = 73) | | |
| --- | --- | --- | --- | --- | --- | --- |
|  | Inactive at 3 month  (n = 72) | Active at 3 month  (n = 67) | *p*-value | Inactive at 3 month  (n = 25) | Active at 3 month  (n = 48) | *p*-value |
| **Age, years** | 64.8 ± 13.4 | 64.0 ± 12.4 | .73 | 64.8 ± 12.5 | 63.2 ± 13.0 | .61 |
| **Male, n (%)** | 47 (65.3%) | 49 (73.1%) | .32 | 19 (76.0%) | 34 (70.8%) | .64 |
| **Stroke type, n (%)** |  |  | .71 |  |  | .26 |
| Ischemic | 63 (87.5%) | 60 (89.6%) |  | 20 (80.0%) | 43 (89.6%) |  |
| Hemorrhagic | 9 (12.5%) | 7 (10.4%) |  | 5 (20.0%) | 5 (10.4%) |  |
| **Cortical involvement, n (%)** |  |  | .72 |  |  | .64 |
| Yes | 29 (40.3%) | 29 (43.3%) |  | 9 (36.0%) | 20 (41.7%) |  |
| No | 43 (59.7%) | 38 (56.7%) |  | 16 (64.0%) | 28 (58.3%) |  |
| **Aphasia, n (%)** |  |  | .24 |  |  | .76 |
| Yes | 7 (9.7%) | 11 (16.4%) |  | 3 (12.0%) | 7 (14.6%) |  |
| No | 65 (90.3%) | 56 (83.6%) |  | 22 (88.0%) | 41 (85.4%) |  |
| **Exercise intervention, n (%)** |  |  | < .001* |  |  |  |
| Yes | 25 (34.7%) | 48 (71.6%) |  | n/a | n/a |  |
| No | 47 (65.3%) | 19 (28.4%) |  | n/a | n/a |  |
| **Past medical history, n (%)** |  |  |  |  |  |  |
| Hypertension | 56 (77.8%) | 49 (73.1%) | .20 | 21 (84.0%) | 36 (75.0%) | .38 |
| Diabetes mellitus | 23 (31.9%) | 19 (28.4%) | .65 | 6 (24.0%) | 14 (29.2%) | .64 |
| Atrial fibrillation | 6 (8.3%) | 11 (16.4%) | .15 | 2 (8.0%) | 8 (16.7%) | .31 |
| Previous stroke | 14 (19.4%) | 10 (14.9%) | .48 | 3 (12.0%) | 9 (18.8%) | .46 |
| **Body mass index, n (%)** |  |  | .069 |  |  | .23 |
| < 18.5 | 5 (6.9%) | 0 |  | 1 (4.0%) | 0 |  |
| 18.5-24.9 | 44 (61.1%) | 40 (59.7%) |  | 16 (64.0%) | 26 (54.2%) |  |
| ≥ 25 | 23 (31.9%) | 27 (40.3%) |  | 8 (32.0%) | 22 (45.8%) |  |
| **Smoking, n (%)** |  |  | .80 |  |  | .52 |
| Current smoker | 18 (25.0%) | 18 (26.9%) |  | 6 (24.0%) | 15 (31.3%) |  |
| Non-smoker | 54 (75.0%) | 49 (73.1%) |  | 19 (76.0%) | 33 (68.8%) |  |
| **Education level, n (%)** |  |  | .28 |  |  | .78 |
| < Elementary school | 13 (18.1%) | 8 (11.9%) |  | 5 (20.0%) | 7 (14.6%) |  |
| Middle school | 7 (9.7%) | 14 (20.9%) |  | 4 (16.0%) | 10 (20.8%) |  |
| High school | 20 (27.8%) | 17 (25.4%) |  | 7 (28.0%) | 10 (20.8%) |  |
| > College | 32 (44.4%) | 28 (41.8%) |  | 9 (36.0%) | 21 (43.8%) |  |
| **Marital status** |  |  | .12 |  |  | .074 |
| Married | 59 (81.9%) | 61 (91.0%) |  | 20 (80.0%) | 45 (93.8%) |  |
| Others | 13 (18.1%) | 6 (9.0%) |  | 5 (20.0%) | 3 (6.3%) |  |
| **Occupation** |  |  | .91 |  |  | .25 |
| Yes | 34 (47.2%) | 31 (46.3%) |  | 10 (40.0%) | 26 (54.2%) |  |
| Others | 38 (52.8%) | 36 (53.7%) |  | 15 (60.0%) | 22 (45.8%) |  |
| **mRS at discharge** |  |  | .006* |  |  | .059 |
| 0-1 | 5 (6.9%) | 18 (26.9%) |  | 4 (16.0%) | 15 (31.3%) |  |
| 2 | 22 (30.6%) | 14 (20.9%) |  | 11 (44.0%) | 9 (18.8%) |  |
| 3 | 45 (62.5%) | 35 (52.2%) |  | 10 (40.0%) | 24 (50.0%) |  |
| **PHQ-9 at discharge** |  |  | .20 |  |  | .34 |
| 0-4 (No depression) | 42 (64.6%) | 30 (49.2%) |  | 10 (43.5%) | 25 (55.6%) |  |
| 5-9 (Mild) | 14 (21.5%) | 17 (27.9%) |  | 9 (39.1%) | 10 (22.2%) |  |
| > 10 (Moderate to severe) | 9 (13.8%) | 14 (23.0%) |  | 4 (17.4%) | 10 (22.2%) |  |
| **Length of stay, days** | 21.3 ± 11.7 | 18.1 ± 8.5 | .077 | 16.6 ± 11.8 | 16.5 ± 7.2 | .97 |

mRS: modified Rankin Scale; PHQ-9: Patient Health Questionnaire-9.

**p* < .05.
