# Supplementary material for: The effect of a telephone-based intervention on physical activity after stroke
Source: PLoS One. 2022 Oct 20;17(10):e0276316. doi: 10.1371/journal.pone.0276316 (PMC9584526; doi:10.1371/journal.pone.0276316)
Supplement: S4 Table — (DOCX) [file pone.0276316.s005.docx]

**S4 Table. The change in mRS scores according to the group (intervention vs. control) or the change in PA.**

|  | **Change of mRS (**between discharge and 3 months) | | |  |
| --- | --- | --- | --- | --- |
|  | Improved | Stationary | Aggravated | *p*-value |
| **Group** |  |  |  | 0.117 |
| Intervention (n = 73) | 49 (67.1%) | 17 (23.3%) | 7 (9.6%) |  |
| Control (n = 66) | 50 (75.8%) | 15 (22.7%) | 1 (1.5%) |  |
|  |  |  |  | 0.833 |
| **Changes in PA in the intervention group**  (between discharge and 3 months) |  |  |  |  |
| Improved (n = 29) | 19 (65.5%) | 6 (20.7%) | 4 (13.8%) |  |
| Stationary (n = 33) | 22 (66.7%) | 9 (27.3%) | 2 (6.0%) |  |
| Aggravated (n = 11) | 8 (72.7%) | 2 (18.2%) | 1 (9.1%) |  |

mRS: modified Rankin Scale; PA: physical activity.
