# Supplementary material for: The effect of a telephone-based intervention on physical activity after stroke
Source: PLoS One. 2022 Oct 20;17(10):e0276316. doi: 10.1371/journal.pone.0276316 (PMC9584526; doi:10.1371/journal.pone.0276316)
Supplement: S5 Table — (DOCX) [file pone.0276316.s006.docx]

**S5 Table. Comparison between the intervention group and excluded participants (readmission, non-responder, and disagreement) admitted in 2020.**

|  | Intervention group  (n = 73) | Excluded participants  (n = 32) | *p*-value |
| --- | --- | --- | --- |
| **Age, years** | 63.7 ± 12.8 | 65.9 ± 13.4 | .43 |
| **Male, n (%)** | 53 (72.6%) | 19 (59.4%) | .18 |
| **Stroke type, n (%)** |  |  | .035* |
| Ischemic | 63 (86.3%) | 22 (68.8%) |  |
| Hemorrhagic | 10 (13.7%) | 10 (31.3%) |  |
| **Past medical history, n (%)** |  |  |  |
| Hypertension | 57 (78.1%) | 23 (71.9%) | .49 |
| Diabetes mellitus | 20 (27.4%) | 14 (43.8%) | .099 |
| Atrial fibrillation | 10 (13.7%) | 5 (15.6%) | .80 |
| Previous stroke | 12 (16.4%) | 6 (18.8%) | .77 |
| **Body mass index, n (%)** |  |  | .004* |
| < 18.5 | 1 (1.4%) | 6 (18.8%) |  |
| 18.5-24.9 | 42 (57.5%) | 16 (50.0%) |  |
| ≥ 25 | 30 (41.1%) | 10 (31.3%) |  |
| **Smoking, n (%)** |  |  | .95 |
| Current smoker | 21 (28.8%) | 9 (28.1%) |  |
| Non-smoker | 52 (71.2%) | 23 (71.9%) |  |
| **Education level, n (%)** |  |  | .89 |
| < Elementary school | 12 (16.4%) | 7 (21.9%) |  |
| Middle school | 14 (19.2%) | 6 (18.8%) |  |
| High school | 17 (23.3%) | 8 (25.0%) |  |
| > College | 30 (41.1%) | 11 (34.4%) |  |
| **Marital status** |  |  | .004* |
| Married | 65 (89.0%) | 21 (65.6%) |  |
| Others | 8 (11.0%) | 11 (34.4%) |  |
| **Occupation** |  |  | .60 |
| Yes | 36 (49.3%) | 14 (43.8%) |  |
| Others | 37 (50.7%) | 18 (56.3%) |  |
| **mRS at discharge** |  |  | .32 |
| 0-1 | 19 (26.0%) | 6 (18.8%) |  |
| 2 | 20 (27.4%) | 6 (18.8%) |  |
| 3 | 34 (46.6%) | 20 (62.5%) |  |
| **mRS at 3 months after discharge** |  |  |  |
| 0-1 |  |  |  |
| 2 |  |  |  |
| 3 |  |  |  |
| **PHQ-9 at discharge** |  |  | .55 |
| 0-4 (No depression) | 35 (51.5%) | 12 (42.9%) |  |
| 5-9 (Mild) | 19 (27.9%) | 11 (39.3%) |  |
| > 10 (Moderate to severe) | 14 (20.6%) | 5 (17.9%) |  |
| **Length of stay, days** | 16.5 ± 9.0 | 17.2 ± 8.5 | .73 |

mRS: modified Rankin Scale; PHQ-9: Patient Health Questionnaire-9.

**p* < .05.
