# Supplementary material for: The effect of a telephone-based intervention on physical activity after stroke
Source: PLoS One. 2022 Oct 20;17(10):e0276316. doi: 10.1371/journal.pone.0276316 (PMC9584526; doi:10.1371/journal.pone.0276316)
Supplement: Attachment — Submitted filename: Response to Reviewers_09.27.docx [file pone.0276316.s007.docx]

**Manuscript ID:** PONE-D-22-17677

**Manuscript title:** The Effect of a Telephone-based Intervention on Physical Activity after Stroke

**#Reviewer 1**

**#Comment 1-1**

Depression is evaluated by PHQ-9, but evaluation of comprehension and memory ability for telephone intervention may affect the results. Therefore, it is desirable to add the location of the lesion (cortical or subcortical) and the presence or absence of aphasia (especially the presence or absence of comprehension disorders) to the comparison of baseline characteristics.

**Response:** We thank the reviewer for this comment. Cortical involvement was determined after reviewing imaging findings. Aphasia was defined based on the NIHSS item 9 for best language during admission. In the fully adjusted model for the odds ratio of the intervention, the cortical involvement and aphasia were also adjusted.

**Changes made in the manuscript:**

*Page 6, lines 102-104 & Page 7, lines 105-106.* After reviewing imaging studies, stroke type and cortical involvement were determined. The presence of aphasia was defined as 1 or more on the National Health Institute Stroke Scale item 9 for best language (range 0 – 3) during the admission.

*Page 7, lines 120-124.* The results were adjusted for age and sex (adjusted ORs) or age, sex, stroke type, cortical involvement, aphasia, a history of hypertension, diabetes mellitus, atrial fibrillation, and stroke, BMI, smoking, education level, marital status, occupation, mRS and PHQ-9 at discharge, and length of stay (fully adjusted ORs).

*Page 8, lines 133-134.* Aphasia was present in 18 patients (12.9%); most were mild non-fluent or anomic type.

**Table 1**

| **Cortical involvement, n (%)** |  |  | .62 |
| --- | --- | --- | --- |
| Yes | 29 (39.7%) | 29 (43.9%) |  |
| No | 44 (60.3%) | 37 (56.1%) |  |
| **Aphasia, n (%)** |  |  | .78 |
| Yes | 10 (13.7%) | 8 (12.1%) |  |
| No | 63 (86.3%) | 58 (87.9%) |  |

**Table 2**

|  | Group | n | Crude OR (95% CI) | *p*-value | Adjusted OR† (95% CI) | *p*-value | Fully adjusted OR‡ (95% CI) | *p*-value |
| --- | --- | --- | --- | --- | --- | --- | --- | --- |
| mRS 0–3  (n = 139) | Intervention | 73 | 4.749 (2.313 – 9.752) | < .001 | 4.676 (2.272 – 9.624) | < .001 | 5.143 (1.837 – 14.400) | .002 |
|  | Control | 66 | Reference | | | | | |
| mRS 2–3 alone  (n = 116) | Intervention | 54 | 4.518 (2.052 – 9.947) | < .001 | 4.547 (2.059 – 10.042) | < .001 | 5.643 (1.823 – 17.467) | .003 |
|  | Control | 62 | Reference | | | | | |
| mRS 3 alone  (n = 80) | Intervention | 34 | 7.636 (2.805 – 20.791) | < .001 | 7.213 (2.625 – 19.818) | < .001 | 23.344 (3.320 – 164.143) | .002 |
|  | Control | 46 | Reference | | | | | |

**#Comment 1-2**

It is unclear whether the band of the curve in Fig.3 shows a continuous change or a discontinuous change. The curve is shown as active to inactive after 1 month, active again after 2 months, and inactive again after 3 months. Is that interpretation correct?

**Response:** We thank the reviewer for this comment. The level of physical activity was measured four times (at discharge and 1, 2, and 3 months after discharge). Fig. 3 (Sankey diagram) illustrates the changes in physical activity level between consecutive time points, which are discontinuous. Therefore, Fig. 3 was revised to reveal the discontinuity clearly, and the sentences about physical activity evaluation have been added.

**Changes made in the manuscript:**

*Page 5, lines 82-84.* PA was monitored through a phone interview (at discharge and 1, 2, and 3 months after discharge), and the target PA level was determined after discussion with the patients.

*Page 6, lines 93-95.* Participants responded to a questionnaire regarding PA via telephone (four times for patients hospitalized in 2020 and once for patients hospitalized in 2019).

*Page 6, lines 98-99.* The change in PA level was classified into improved, stationary, and aggravated in the intervention group.

**#Comment 1-3**

It would be better to evaluate and analyze changes in the degree of PA (Health enhancing PA/Minimally active).

**Response:** Thank you for your comment. Few patients displayed health-enhancing PA as illustrated in the revised Fig 3 (B) (Green = Health-enhancing PA, Orange = Minimally active, and Red = Inactive). The number of patients with health-enhancing PA was 7, 0, 2, and 6 at discharge, 1, 2, and 3 months after discharge, respectively. Therefore, it was challenging to analyze the clinical characteristics of those with health-enhancing PA separately. Instead, we revised Fig 3 to include changes in PA degree ((A): active and inactive, (B): health-enhancing physical activity, minimally active, and inactive).

**Changes made in the manuscript:**

**Fig 3. Changes in physical activity ((A): Active and Inactive, (B): Health-enhancing physical activity (HEPA), Minimally active, and Inactive) in the intervention group from discharge to follow-up at 3 months.**

**#Comment 1-4**

Although the purpose of this study is the effect of a telephone-based intervention on PA, it is desirable to consider the relationship between PA improvement and mRS improvement in the discussion.

**Response:** Thank you for your comment. Baseline mRS scores differed between the intervention and control groups; however, mRS score improvement within 3 months after discharge was not significantly different between both groups. In addition, mRS score improvement did not significantly differ based on PA changes in the intervention group (the analysis was conducted only in the intervention group because the PA level at discharge was not evaluated in the control group). This may suggest that PA improvement results from the intervention rather than PA improvement during stroke recovery. The table is presented as S4 Table.

**Changes made in the manuscript:**

**S4 Table. The change in mRS scores according to the group (intervention vs. control) or the change in PA.**

|  | **Change of mRS (**between discharge and 3 months) | | |  |
| --- | --- | --- | --- | --- |
|  | Improved | Stationary | Aggravated | *p*-value |
| **Group** |  |  |  | 0.117 |
| Intervention (n = 73) | 49 (67.1%) | 17 (23.3%) | 7 (9.6%) |  |
| Control (n = 66) | 50 (75.8%) | 15 (22.7%) | 1 (1.5%) |  |
|  |  |  |  | 0.833 |
| **Changes in PA in the intervention group**  (between discharge and 3 months) |  |  |  |  |
| Improved (n = 29) | 19 (65.5%) | 6 (20.7%) | 4 (13.8%) |  |
| Stationary (n = 33) | 22 (66.7%) | 9 (27.3%) | 2 (6.0%) |  |
| Aggravated (n = 11) | 8 (72.7%) | 2 (18.2%) | 1 (9.1%) |  |

mRS: modified Rankin Scale; PA: physical activity.

*Page 12, lines 169-172.* When the change in mRS score between discharge and 3 months was divided into improved, stationary, and aggravated, no significant difference was observed between the intervention and control groups. In the intervention group, the change in PA did not significantly differ based on changes in mRS scores (S4 Table).

*Page 13, lines 189-192.* In addition, changes in the level of PA did not significantly differ based on changes in mRS scores of the intervention group, suggesting that an increase in PA would result from the intervention rather than improved mRS scores during stroke recovery.

**#Reviewer 2**

**#Comment 2-1**

The authors present an important topic of nurse intervention and telephone monitoring of the health and physical activity of patients who have recently had a stroke. The authors rightly noted that physical activity provides benefits for health-related outcomes after stroke and cardiovascular risk factor management. Physical activity also supports social participation, helping stroke survivors to achieve their goals and adjust to life after stroke.

Of course, telephone interventions limit the amount of information that can be gathered about a patient's current condition, but how previous studies have shown the feasibility and effectiveness of telephone-based interventions in patients after stroke. The study is designed well. The title and abstract cover the main aspect of the work. The introduction provides background and information relevant to the study. The methodology are described very exactly and clearly. The statistical analysis is performed correctly. The results are described accurately and clearly. The authors accurately described the limitations of the study.

**Response:** We thank the reviewer for this comment. We agree with the comment and believe that our study, consistent with previous studies, supports the telephone-based intervention on physical activity improvement in patients with stroke. Thank you.
